# Improved *Macaca fascicularis* gene annotation reveals evolution of gene expression profiles in multiple tissues

**DOI:** 10.1186/s12864-018-5183-y

**Published:** 2018-11-01

**Authors:** Tao Tan, Lin Xia, Kailing Tu, Jie Tang, Senlin Yin, Lunzhi Dai, Peng Lei, Biao Dong, Hongbo Hu, Yong Fan, Yang Yu, Dan Xie

**Affiliations:** 1 Department of Obstetrics and Gynecology, Peking University Third Hospital, Beijing, China; 2 State Key Laboratory of Biotherapy & Collaborative Innovation Center for Biotherapy, West China Hospital, Sichuan University, Chengdu, China; 3 Center of Precision medicine, West China Hospital, Sichuan University, Chengdu, China; 4 Lab of PTM and Department of General Practice, West China Hospital, Sichuan University, Chengdu, China; 5 West China School of Basic Medical Sciences & Forensic Medicine, Sichuan University, Chengdu, China; 6 National Clinical Research Center for Geriatrics, West China Hospital, Sichuan University, Chengdu, China; 7 Department of Rheumatology and Immunology, West China Hospital, Sichuan University, Chengdu, China; 8 Key Laboratory for Major Obstetric Diseases of Guangdong Province, The Third Affiliated Hospital of Guangzhou Medical University, Guangzhou, China; 9 Yunnan Key Laboratory of Primate Biomedical Research, Institute of Primate Translational Medicine, Kunming University of Science and Technology, Kunming, 650500 Yunnan China

**Keywords:** Crab-eating macaque, Evolution, Gene regulation

## Abstract

**Backgrounds:**

*Macaca fascicularis* (*M. fascicularis*) is a primate model organism that played important role in studying human health. It is vital to better understand the similarity and differences of gene regulation between *M. fascicularis* and human. Current comparative study of gene regulation between the two species are limited by low quality of gene annotation and lack of regulatory element data on *M. fascicularis* genome.

**Results:**

In this study, we improved the *M. fascicularis* gene annotation with 57 gene expression data from multiple tissues and, more importantly, a manual curation procedure. The new annotation enabled us to map gene expression and identify gene location more accurately.

**Conclusions:**

Comparing with human gene expression data from the same cell types, we characterized the evolution of expression patterns of homologous genes.

**Electronic supplementary material:**

The online version of this article (10.1186/s12864-018-5183-y) contains supplementary material, which is available to authorized users.

## Backgrounds

*Macaca fascicularis (M. fascicularis)* is also called the cynomolgus, long tailed macaque or Crab-eating macaque. It is a primate model organism that played important role in the study of infectious diseases [1–3], neurobiology [4–6], metabolism [7–9], hemopoietic system [10, 11] and embryonic stem cells [12, 13]. Because of their phylogenetic closeness to human, *M. fascicularis* is also widely used in pharmaceutic studies [14–16]. Therefore, it is important to better understand the similarity and differences between *M. fascicularis* and human species. Previous studies have reported the evolution of the genome sequence [17] and gene expression [18, 19] between the two species. However, to date, the study of evolution between the two species is still insufficient, especially at gene regulatory level.

One important reason for inaccurate comparison is the lack of good gene annotation on *M. fascicularis* genome. Currently, the human genome has the best gene annotation due to large research community. On one hand, human species has collected the most comprehensive transcription data; On the other hand, dedicated consortia, such as Genecode project [20, 21], have made good efforts to manually curate the annotation. In contrast, the transcription dataset for *M. fascicularis* is limited and no manual curation has been performed [19, 22]. The most up to date gene annotation for *M. fascicularis* were *M. fascicularis* Annotation Release 101 from NCBI [23] and *M. fascicularis* Annotation Release 91 from Ensembl [21]. Both were based on small transcription dataset and were only annotated using computational pipeline.

In this study, we generated RNA-seq data from multiple *M. fascicularis* tissues and improved the gene annotation combining computational pipeline and manual curation. The improved genome annotation had more precise transcription starting sites and enabled us to estimate gene expression levels more accurately. Combining RNA-seq data from the same tissues and cell types, we revealed the conserved and evolved pattern of gene expression between *M. fascicularis* and human homologous genes.

## Results

### The generation and assembly of data resources for the gene annotation of *M. fascicularis* genome

To better annotate the genes on the *M. fascicularis* genome, we generated 29 RNA-seq datasets and collected 28 existing RNA-seq datasets [19] (methods) (Additional file 1: Figure S1a, Additional file 2: Table S1). The combined RNA-seq dataset comprehensively represented the expression profile of 24 tissues/cell-types encompassing 8 main systems, including four digestive system organs(colon, rectum, stomach, liver), seven hematopoietic or immune system tissues or cell types (bone marrow, lymph node, spleen, thymus, CD4+, CD8+, CD14+), four nervous system tissues (cerebellum, frontal cortex, pituitary, temporal lobe), three reproductive system organs (epididymis, prostate, testis), two urinary system organs (kidney, bladder), two circulatory system tissues (heart and postcava) and two major organs of respiratory system and motor system (lung and skeletal muscle). All the RNA-seq libraries were generated using “ribosomal depletion” technology (methods), which better represented full-length mRNA transcripts and long non-coding RNA transcripts. We processed the RNA-seq data with a unified computational pipeline (methods). A total of ~ 7.1G uniquely mapped reads (76.16 MB~ 193.16 MB, median = 122.00 MB) were included in the following annotation procedure (Additional file 2: Table S1).

To make the gene annotation most up-to-date, in addition to RNA-seq data, we also downloaded the latest 38,433 cDNA sequences of *M. fascicularis* from Pre Ensembl (average length of 928 bp, ranging from 44 bp to 61,704 bp) [24, 25], and 172,829 EST sequences of *M. fascicularis* from UCSC genome browser (average length of 663.2 bp, ranging from 29 bp to 1206 bp). For protein sequence dataset, due to the fact that there were only 15 reviewed protein sequences of *M. fascicularis* in Uniprot and the protein sequences have higher sequence conservation than nucleotide sequences in evolution, we downloaded 23,645 known protein sequences of 9 primates (*Homo sapiens*, *Macaca mulatta*, *Pan troglodytes*, *Pongo abelii*, *Chlorocebus sabaeus*, *Papio Anubis*, *Gorilla gorilla gorilla*, *Nomascus leucogenys* and *M. fascicularis*) for more accurate gene prediction (Additional file 3: Table S2).

### More complete and accurate gene annotation achieved from the combined computational pipeline and manual curation

**Fig. 1:**
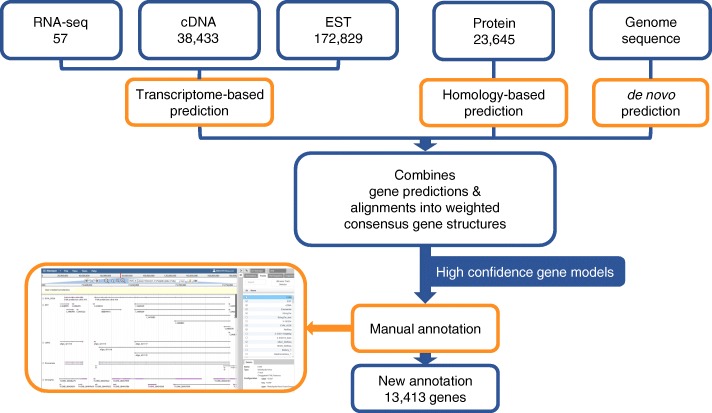
Workflow of annotation procedure. Overview of the data and workflow of the computational annotation and manual annotation

To comprehensively use all the data resource we assembled to achieve a more complete and accurate gene annotation, we designed an annotation procedure that combined automated computational pipeline and manual curation (Fig. 1). We developed the automated computational annotation pipeline using a collection of carefully evaluated software and in-house scripts [21, 26].

In short, the core computational procedure consisted of two phases. Phase I (prediction phase) contained three separate gene prediction procedures, which took into consideration of genome sequence data, protein sequence data, and long transcript data (EST, cDNA, assembled RNA-seq data) respectively. Phase II (consensus phase) combined the gene prediction results from Phase I using a weighted consensus strategy.

Among the three gene prediction procedures of Phase I, the first process was de novo gene prediction from *M. fascicularis* genome sequences. Considering sensitivity, specificity, and accuracy, we chose five complementary software, including Augustus [27], GENSCAN [28], GeneMark [29], Glimmer-HMM [30], and SNAP [31], in this process. In the second process, we used exonerate [32] to align know protein sequence to *M. fascicularis* genome. A total of 23,645 protein sequences of *M. fascicularis* and 8 closely related species were aligned and produced 6,259,610 alignments from this process. The third process predicted genes based on the alignment of EST, cDNA and RNA-seq reads. In this process, 172,829 alignments were produced from ESTs; 264,372 alignments were produced from cDNAs; 552,322 transcripts were assembled from RNA-seq reads.

In phase II, we first used EVM (EVidenceModeler) [33] to construct a set of weighted consensus gene structures by combining the predicted gene structures from the three prediction procedures described above; and then updated the consensus gene structures with cDNAs alignments (adding UTRs, adjusting exon boundaries and establishing models of alternative splicing) using PASA [34]. after Phase II, a total of 23,282 genes with average length of 43,579 bp were annotated as the candidate reference gene model. The computational annotated gene model covered 33.94% of euchromatin and chrX of Crab-eating monkey genome. A total of 17,774 candidate genes were predicted as coding genes by Coding-Potential Assessment Tools (CPAT) [35] and Coding Potential Calculator (CPC) [36]. Among the predicted gene models, 15,684 (67.37%) were supported by at least two different biological evidences, which were classified as high confidence genes.

To improve the accuracy of the gene annotation, we manually curated the gene models predicted by our automated computational pipeline. We assigned gene models into 4 confidence levels. Level one (highest confidence) gene models were confirmed by refseq annotation and at least two independent pieces of evidences (transcript sequence match, protein sequence match, or RNA-seq data match); Level two gene models were refseq annotation only; Level three gene models were confirmed by at least two independent pieces of evidences but absent from refseq annotation; Level four (lowest confidence) gene models were confirmed by only one piece of evidence. A total of 2006; 77; 13,691; and 11,345 gene models were assigned to the four levels, respectively. We then manually curated the gene models following a guideline that was included in the (Additional file 4: Figure S2) (methods). In general, we manually aligned and adjusted the start and end location of each exon, TSS, TES, 5’UTR, and 3’UTR.

In the end, a total of 13,413 genes passed the manual curation criteria, which covered 28.09% of all autosome and X chromosome of *M. fascicularis* genome (chrY and chrM were not annotated due to poor reference genome quality) (Fig. S1b). The newly annotated genes were 62,503 bp in average. In total, 771,632 exons with average length of 615 bp were included. We used Coding Potential Calculator (CPC) [36] and Coding-Potential Assessment Tools (CPAT) [35] to predict the coding potential of the newly annotated genes. Totally 13,196 genes were marked as “coding gene” by both tools.

**Fig. 2:**
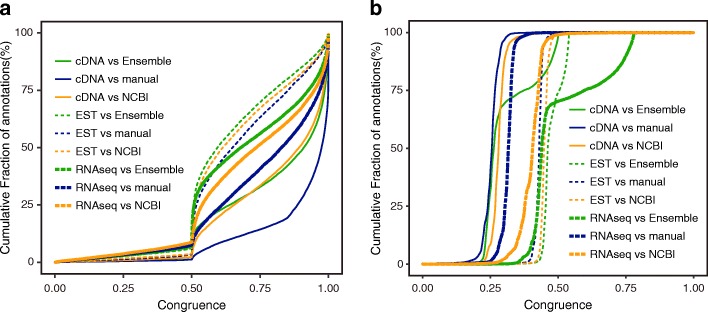
Quantitative measure of the comparison of different annotation versions. **a** The cumulative congruence distributions of Ensemble *Macaca fascicularis* 5.0.91 annotation, NCBI *Macaca fascicularis* release 101 and the new annotation (manual) on gene level, compared with cDNA alignments, EST alignments and RNA-seq alignments. Great congruence means high strong consistency with evidence. **b** The cumulative congruence distributions of Ensemble *Macaca fascicularis* 5.0.91 annotation, NCBI *Macaca fascicularis* release 101 and the new annotation on exon level, compared with cDNA alignments, EST alignments and RNA-seq alignments. Great congruence means high strong consistency with evidence

We evaluated the quality of the newly annotated gene models using congruency (methods) between the annotation and evidences (cDNA, EST, and RNA-seq reads alignment) [37]. At full gene length level, the congruency between the newly annotated gene model and evidences were significantly higher than between current NCBI annotation and evidences (Fig. 2a, Additional file 5: Figure S3a, methods). Likewise, at exon level, though both the newly annotated gene models and NCBI annotation had high congruency (> 0.5), the new annotation outperformed NCBI annotation with all three types of evidences (Fig. 2b, Additional file 5: Figure S3b, methods). We summarized the statistics of our gene annotation and compared them with the other annotations in Table 1.

**Table 1:** Comparision of different annotation

Future		Human Gencode version 19	Refseq	NCBI 101 (2016.02)	Ensemble version1 (2017.12)	Computational annotation	Manual Annotation
Genes		57,820 (main chromosome without chrY)	-	33,368 (31,950 main chromosome without chrY)	28,592 (27,966 main chromosome without chrY)	23,282 (main chromosome without chrY)	13,413 (main chromosome without chrY)
Protein-coding genes		20,345	-	20,627	21,404	17,774	13,196
average length of genes		29,907 bp	-	41,827 bp	38,598 bp	43,579 bp	62,503 bp
transcripts		196,520	2037	76,559	53,156	27,029	-
average length of transcripts		34,243 bp	40,127 bp	80,039 bp	49,987 bp	44,243 bp	-
Number of transcripts per gene		3.4	1	2.29	1.86	1.16	-
CDSs		269,043	15,307	62,934	481,660	27,628	-
average length of CDSs		159 bp	147 bp	709 bp	155 bp	1410 bp	-
Exons		562,673	16,597	289,695	500,876	201,670	771,632
average length of exons		330 bp	217 bp	403 bp	218 bp	195 bp	615 bp
Number of exons per transcript		2.86	8.15	3.78	9.42	7.46	13.93
coverage rate(main chromosomes)	min	12.08%	1.08%(without chrY, chrM)	33.87%(without chrY)	27.45%(without chrY)	23.95%(without chrY, chrM)	18.14%(without chrY, chrM)
	median	51.71%	3.07%(without chrY, chrM)	48.96%(without chrY)	39.94%(without chrY)	35.17%(without chrY, chrM)	29.96%(without chrY, chrM)
	mean	51.17%	2.91%(without chrY, chrM)	48.14%(without chrY)	40.79%(without chrY)	35.92%(without chrY, chrM)	29.22%(without chrY, chrM)
	max	92.50%	4.59%(without chrY, chrM)	68.36%(without chrY)	92.42%(without chrY)	50.57%(without chrY, chrM)	39.45%(without chrY, chrM)
coverage rate (genome, all main chromosomes)		50.39%	2.81%(without chrY, chrM)	46.8%(without chrY)	37.98%(without chrY)	33.94%(without chrY, chrM)	28.09%(without chrY, chrM)

### The evolution rate of gene expression profiles in multiple tissues between *M. fascicularis* and human were different

The newly curated gene annotation empowered us to map the gene expression levels more accurately, and, therefore, to study the evolution of gene expression profiles between *M. fascicularis* and human more reliably. We identified 11,446 one-to-one orthologous genes between human and *M. fascicularis* (Additional file 6: Table S3) (methods). We found many genes that associated with human disease in this orthologous gene list. For example, we found Alzheimer’s Disease risk genes APOE, PLD3, TREM2, UNC5C, AKAP9, and ADAM10 in our orthologous gene list [38]. Likewise, genes related with other disease such as epilepsy, Schizophrenia, HIV infection and multiple kinds of tumor were also included in the list (Additional file 6: Table S3). More complete sequence annotation of these genes will be helpful to the development and improvement of human disease model of *M. fascicularis*.

For comparison, we included RNA-seq data from 13 human tissues generated by three labs [37, 39, 40] (Additional file 7: Table S4). There were 54 sets of human RNA-seq data. They associated with 45 individual, 15 male, 16 female and 14 unknowns in sex. A total of ~ 4.7GB (30.2 MB~ 282.4 MB, median = 86.7 MB) uniquely mapped reads were included in the human data set. Both human and *M. fascicularis* showed high correlation in RNA expression within each tissue (Additional file 8: Figure S4a).

**Fig. 3:**
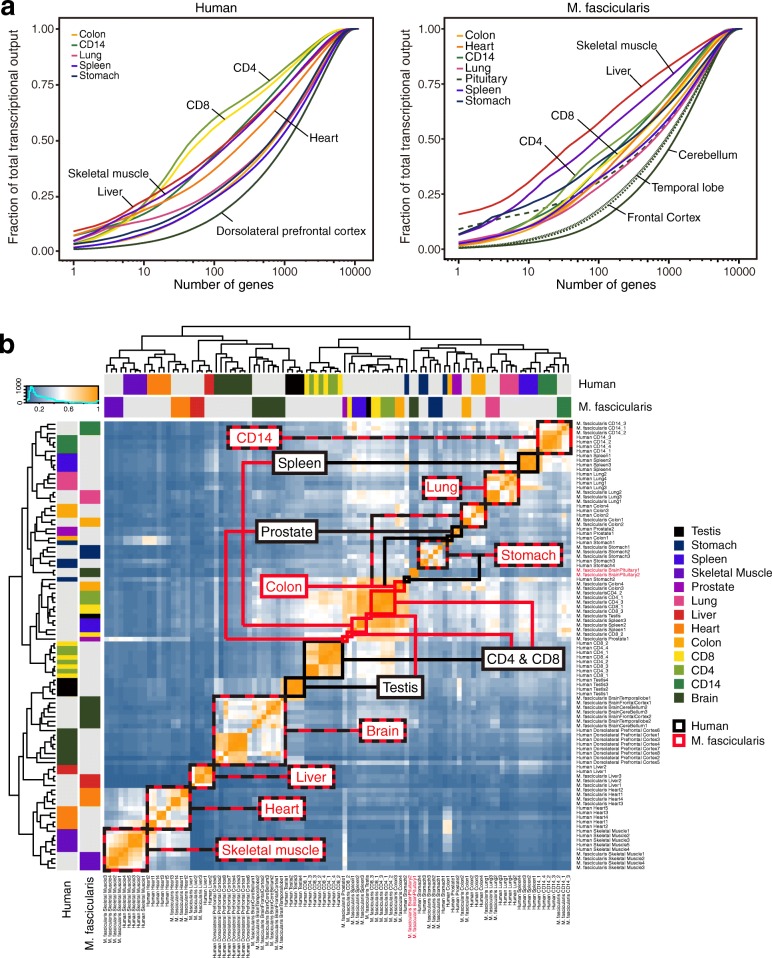
Complexity of tissue transcriptomes and comparison of tissue expression profiles across human and *M. fascicularis*. **a** Cumulative distribution of the faction of total orthologous transcription contributed by genes which in order of decreasing expression in each tissue (x axis). Left panel shows complexity of tissue transcriptomes in human, right panel shows *M. fascicularis*. **b** The heat map shows the all-versus-all Pearson correlation matrix between 13 tissues in human and *M. fascicularis* over all 11,446 orthologous genes. Red box means specific tissue expression pattern of *M. fascicularis*; Black box presents specific tissue expression pattern of human; black and red box presents similar tissue expression pattern of same kind of tissue between human and *M. fascicularis*. Orange means highest correlation coefficient, blue means lowest correlation coefficient. Samples from pituitary of *M. fascicularis* were colored in red

We first explored the expression patterns of the orthologous genes in these 13 tissues between human and *M. fascicularis* (methods). Previous study has shown that a few tissue-specific genes contribute to more than 50% of total transcript in each tissue, and the complexity of the transcriptome composition are varied among tissues [41]. We plotted the complexity of transcriptome composition in both *M. fascicularis* and human using our data (Fig. 3a). In both species, the transcriptome composition of liver and skeletal muscle had low complexity, where about 100 genes contributed more than 50% of the total transcripts; Whereas tissues from brain cortex had the highest complexity (dorsolateral prefrontal cortex in human data; frontal cortex, temporal lobe, and Cerebellum in *M. fascicularis* data), which agrees with previous findings. Interestingly, the transcriptome composition of CD4 and CD8 cells showed different complexity patterns between *M. fascicularis* and human, where in human they were among the lowest complexity but in *M. fascicularis* they showed high complexity, suggesting higher degree of evolution in these cell types. These results validated that transcriptome signature confers tissue identity in *M. fascicularis* as it does in human and baboon [41–43].

To further examine the similarities between human and *M. fascicularis* in detail, we clustered the tissues of both species based on the expression levels of all the orthologous genes. In total, 8 out of 13 tissues (heart, skeletal muscle, stomach, colon, lung, liver, brain, and CD14 cells) exhibited “tissue dominated clustering” where the same tissue of both species clustered together, indicating highly conservative regulatory programs in these tissues. In contrast, the other 5 tissues (testis, prostate, spleen, CD4 and CD8 cells) were clustered by species, indicating faster evolution of gene expression (Fig. 3b). It is reasonable to see that pituitary from *M. fascicularis* and stomach from both species were clustered together. Because pituitary and stomach are derived from the endoderm [44] and their main function is secretion, which is different from brain cortex. We next looked into the differentially expressed genes between brain cortex and tissues consisting of pituitary and stomach. Unsurprisingly, 1499 orthologous genes (Wilcoxon Rank Sum test, *p*-value < 0.05, mean fold change > 2) that had higher expression levels in brain cortex were enriched on synapse related GO terms, like trans synaptic signaling, chemical synaptic transmission (Additional file 8: Figure S4c). And 1852 pituitary and stomach high expressed orthologous genes (Wilcoxon Rank Sum test, *p* < 0.05, mean fold change > 2) were mainly enriched in the GO term that associated with protein synthesis (Additional file 8: Figure S4d), which is a mainly function of glandular epithelium tissues.

It has been reported that the reproductive systems and immune systems between human and non-human primate evolve faster compared with the other tissues [45–47]. Our results resonant pervious findings, but, on the other hand, suggested different linages of blood cell types had varied evolutionary rate in gene expression.

## Discussion

Animal models have played important role in understanding human health. Non-human primate were animal models most close to human evolutionarily, but still maintain species specific traits. It is, therefore, vital to fully understand the cellular difference between these two species. In this study, we aimed to explore the evolution between *M. fascicularis*, a widely used primate model, and human.

One big obstacle for this study was the poor annotation of *M. fascicularis* genome. There were two key factors for high quality gene annotation: comprehensive transcriptome datasets, and manual curation. Comparing with human genome, which has the highest gene annotation quality, the *M. fascicularis* gene annotation was based on very limited transcriptome datasets and lacked manual curation process. We addressed this problem by generating 29 RNA-seq data from multiple tissues, which doubled the amount of published *M. fascicularis* RNA-seq dataset. Importantly, we also added manual curation to the gene annotation process, which significantly improved the quality of gene annotation with more accurate TSS, TES, and boundary between exon and introns. Although largely improved, our annotation is far from perfect. For example, high quality transcriptome isoform annotation was not feasible due to the lack of full-length transcriptome data. The improvement of gene annotation quality needs continuous efforts. With the advance of new sequencing technology, such as Pacbio or Oxford nanopore, the quality of *M. fascicularis* genome annotation can be further improved.

## Conclusions

We studied the evolution at transcription level, with our new *M. fascicularis* gene annotation, we revisited the comparison of gene expression levels at multiple tissues between *M. fascicularis* and human species. When put the data from two species together, we found two clustering patterns. For some tissues, samples from the two species were clustered together, suggesting more conserved gene expression pattern cross species; for the other tissues, samples from the two species were clustered separately, suggesting more species-specific gene expression patterns. This insight is useful for following up study design when using *M. fascicularis* as model to human. Although the *M. fascicularis* RNA-seq data were sampled from 4 animals, but there’s little doubt that this large RNA-seq data helped us learned the similarities and differences between 13 tissues of human and *M. fascicularis* for the first time. We can learn more about the evolution of transcriptome between human and *M. fascicularis* by using more data of samples that had ages and sexes balanced.

## Methods

### Animals and samples collection

Adult healthy *M. fascicularis* were housed in individual cages at Yuanxi Biotech Inc. Guangzhou and used in this study. All animal procedures were approved by the Institutional Animal Care and Use Committee at Yuanxi Biotech Inc. Guangzhou (YXSW-2016-01). There are twenty different tissues samples from a male *M. fascicularis* who euthanatized were used for transcriptome sequencing. Samples from lung, liver, testis, kidney,a total of 20 tissues were collected from a 4-year-old male *M. fascicularis* whereas CD4+, CD8+ and CD14+ lymphocytes respectively collected from three *M. fascicularis* (15-year-old, 16-year-old, 16-year-old) for transcriptome sequencing. The details of each *M. fascicularis* were listed in Additional file 9: Table S5. For the transcriptome sequencing, We separately used magnetic cell sorting to isolated CD4+, CD8+ and CD14+ lymphocytes from Peripheral Blood Mononuclear Cell (PBMC) of using the MACS® separation (Miltenyi Biotec) according to the manufacturer’s instructions.

### Anesthesia and euthanasia methods

*M. fascicularis* were euthanized according to SOP in Yuanxi Biotech Inc. Guangzhou used for the RNA sequence studies. Briefly, the animals were anesthetized by intraperitoneal administration of pentobarbital (100 mg/kg) and transcardially perfused with 1 L of chilled 0.01 M PBS (pH 7.4, Dulbecco’s phosphate-buffered saline, Sigma-Aldrich, St. Louis, MO) to wash the blood out from the brain tissue. *M. fascicularis* born and raised at the Yuanxi Biotech Inc. Guangzhou monkey house with family group of 20–25, providing a natural illumination and normal social environment. All procedures were approved by the Institutional Animal Care and Use Committee (IACUC) at Yuanxi Biotech Inc. Guangzhou.

### LncRNA library preparation process, RNA quality examination and sequencing

RNA degradation and contamination were detected by 1% agarose gels; RNA purity was checked using the kaiaoK5500®Spectrophotometer (Kaiao, Beijing, China).

RNA integrity and concentration were assessed using the RNA Nano 6000 Assay Kit of the Bioanalyzer 2100 system (Agilent Technologies, CA, USA).

Library preparation for LncRNA sequencing. A total amount of 3 μg RNA per sample was used as initial material for the RNA sample preparations. Ribosomal RNA was removed using Epicentre Ribo-ZeroTM Gold Kits (Human/Mouse/Rat) (Epicentre, USA). Subsequently, the sequencing libraries were generated following manufacturer recommendations with varied index label by NEBNext® UltraTM Directional RNA Library Prep Kit for Illumina (NEB, Ispawich, USA). The details of library construction showed as follow: Firstly, ribosomal RNA was removed by kits, RNA fragmentation and short RNA strands were carried out by NEBNext First Strand Synthesis Reaction Buffer under elevated temperature. Subsequently, First cDNA strand was synthesized using random hexamer primers and RNA fragments as template. Second strand cDNA synthesis was subsequently performed using buffer, dNTPs, DNA polymerase I and RNase H. The library fragments were purified with QiaQuick PCR kits and elution with EB buffer, then terminal repair, add poly(A)and adapter were implemented. In order to select cDNA fragments of preferentially 300 bp in length, the library fragments were purified with agarose gel electrophoresis and the UNG enzyme was used to digest second strand of cDNA. PCR was performed, aimed products were retrieve by agarose gel electrophoresis, and the library was completed. The HiSeq PE (Paired-End) Cluster Kit v4 cBot reagents were used for the cBot cluster amplification system, and libraries were sequenced on Illumina HiSeq X-10.

### RNA-seq raw data alignment and assembly

For *M. fascicularis*, all 57 RNA-seq datasets were aligned to Macaca_fascicularis_5.0 (macFas5, downloaded from UCSC genome browser) with RefSeq annotation via spliced aligner HISAT2 (version 2.0.4) [48] with default parameters. The RNA-seq alignments were assembled using Stringtie (version 1.3.0) [49]. In order to choose the most suitable parameters of Stringtie for each dataset to avoid overlong false exons being assembled, we ran the Stringtie on each RNA-seq data 14 times with different -c parameter (from 2.5 to 15.5 at intervals of 1), which limits the minimum read coverage allowed for transcript assembly, to find out the reads coverage work best for each data. For every iteration, assembled transcript model was compared with RefSeq annotation. The Sensitivity of each iteration was calculated with the formula:

$$ \mathrm{Sensitivity}=\frac{DE}{MDE} $$

Where MDE represents the count of exon detected with default StringTie parameter (−c 2.5). DE represents the count of detected RefSeq exons having more than 90% overlap with newly assembled exons.

Considering intron pollution, the count of detected RefSeq exons which had less than 10% intersection with RefSeq annotated non-exon region was defined as PDE. And exons detection precision was calculated as:

$$ \mathrm{Precesion}=\frac{PDE}{DE} $$

We chose optimal -c parameter for each sample when exon precision get closest to exon sensitivity.

For human, all 54 RNA-seq datasets (Additional file 7: Table S4) were aligned and assembled to human Gencode version 19 annotation (downloaded from UCSC genome browser) via HISAT2 (version 2.0.4) [48] and Stringtie (version 1.3.0) [49], separately, with default parameters.

### Computational annotation

De novo gene predictions of *M. fascicularis* generated by Genemark [29], GlimmerHMM [30], SNAP [31], AUGUSTUS [27] and Genescan [28]. In detail, Genemark predicted genes base on macFas5 using self-training model. SNAP was on human gene annotation (Gencode v19). The precompiled parameter files of human from GlimmerHMM website were utilized as training files when using GlimmerHMM to predict genes. Result of AUGUSTUS (version 3.1) and Genescan were downloaded from UCSC genome browser.

57 RNA-seq datasets of 24 *M. fascicularis* tissues or cells were mapped and assembled by HISAT2-Stringtie workflow (mentioned above) for RNA-based gene structure annotation. All RNA-seq alignments were merged using Cuffmerge.

EST and cDNA sequences were aligned to MacFas5.0 genome by Program to Assemble Spliced Alignments (PASA) [34] with default parameters.

Reviewed Protein sequences of 9 primates (Additional file 3: Table S2) downloaded from Uniport protein database were aligned to MacFas5.0 genome using exonerate [32].

EVidenceModeler (EVM [33]) was utilized to integrate all gene sets mentioned above with different weight scores (1 for de novo gene predictions, 10 for cDNA alignment and 5 for other alignments) and consensus gene models. All candidate gene models were then updated with cDNA alignments by PASA, to correct exon boundaries, adding UTRs, and model for alternative splicing.

### Manual annotation guidelines

Evidences were utilized according to the following priorities: cDNA alignments > EST alignments > high confidence RNA-seq alignments = NCBI annotation = RefSeq annotation > protein alignments = other-species RefSeq genes (downloaded from UCSC table browser). RNA-seq alignments those presented in at least 2 different tissues or cells were considered as high confidence RNA-seq alignments.

#### Annotation guidelines for level one (highest confidence) gene models

Gene models with over 70% of length supported by RefSeq annotation and at least 2 kinds of evidences, were identified as level one gene models. Manual annotations of these genes were shown below:

##### Identical gene model

Gene models would be saved completely if they shared the same gene structure with corresponding gene models in RefSeq annotation.

##### Adjusting the exon of 5′ or 3′ end of a gene model

Divergence between level one gene model and corresponding RefSeq gene model only appeared on the 5′ or 3′ end. The longest 5′ or 3′ end among RefSeq gene model, EVM gene model, cDNA alignments, EST alignments and high confidence RNA-seq generated gene model would be kept in the final gene structure.

##### Gene models with different gene structure

Once gene models did not share the same gene structure with corresponding RefSeq genes, the one supported by the largest amount of evidences (cDNAs, ESTs or RNA-seq alignments) would be saved. If no gene model can reach this criterion the RefSeq gene model would be saved.

##### Gene models overlap with multiple RefSeq gene models

When multiple RefSeq gene models share part of gene structure with one level 1 gene model, we queried their ID on NCBI Gene to find out more details. If those RefSeq gene models were different transcripts of one gene, they would be merged together. Else, we only considered the one supported by most evidences for further adjustment (see 1.1–1.3).

#### Annotation guidelines for level two gene models

Gene models supported by only RefSeq annotation were identified as level two gene models. Mannual annotations of these genes were shown below:

##### RefSeq gene models with duplicate gene ID

Only retained the one which was defined as “best RefSeq” in NCBI database for further adjustment.

##### Single exon RefSeq gene model

Single exon RefSeq gene models would be deleted unless at least one kind of evidences supported such model.

##### Multi-exon RefSeq gene models

Exons would be merged if there were evidences supported such Mergence. When all kinds of evidences supported the gene structure of the RefSeq gene model, it would be saved without modification. More adjustments refer to 3.1–3.5.

#### Annotation guidelines for level three gene models

*Gene models having no overlap with RefSeq gene models were identified as level three gene models.* Manual annotations of these genes were shown below:

##### Adjusting the exon of 3′/5′ end of a gene model

The longest 3′/5′ end among level three gene model, cDNA alignments, EST alignments and related high confidence RNA-seq alignments were kept in the final gene structure.

##### Exon addition

Exons were added into level three gene models if they meet any of the following criteria: *(1) At least one evidence (cDNA alignments, EST alignments and high confidence RNA-seq alignments) supported to add exon in the gene model; (2) At least one gene model of NCBI annotation and RefSeq genes of other-species supported the same exon addition; (3) At least 2 evidences (cDNA alignments, EST alignments and high confidence RNA-seq alignments) supported the same exon addition, we would add the exon into the corresponding gene model.*

##### Exon deletion

We deleted exons those without any biological evidences support. Once the exon of 5′ or 3′ end of a gene model had been deleted, referring to 3.1 to adjust the length of newly exon of 5′ or 3′ end.

##### Gene model replacement

*Once level three gene model had different gene structure with corresponding cDNA alignments*, it would be replaced by the longest cDNA alignment that was supported by other evidence (EST and confidence RNA-seq alignments).

##### Mergence and separation

If all kinds of evidences supported mergence or separation, then we merged or split those gene models or exons referred to the corresponding cDNA alignments and EST alignments.

##### Different isoforms of a gene model

When multiple gene models located at one loci, and the overlap rates between each pair of them exceeded 70%, then we considered they are different isoforms of one gene model and merged them together.

### Quantitative measure of the comparison of different annotation versions

Congruency (C, the average of sensitivity and specificity) was used to evaluated the performance of gene annotation with the following formula [50]:

$$ \mathrm{C}=\left(\mathrm{SN}+\mathrm{SP}\right)/2 $$

Where SP means specificity, and SN means sensitivity. For a given gene model i and evidence j (cDNA alignments, EST alignments and RNA-seq alignments), the gene level sensitivity was calculated with the formula:

$$ \mathrm{SN}=\left|i\cap j\left|/\left|j\right.\left|\right.\right.\right. $$

And the specificity was calculated with the formula:

$$ \mathrm{SP}=\left|i\cap j\right|/\left|i\right| $$

Where *i* ∩ *j* means the number of base pair of *i* and *j*, | *i* | and | *j* | represents the length of gene model *i* and evidence *j*, respectively. For exon level, we used the same formula to calculate the congruency of different annotation version with different evidence, where *i* represents exons annotated in annotation, and *j* was overlapped exons of evidences assembled in cDNA alignments, EST alignments or RNA-seq alignments.

### Protein coding potential and one-to-one orthologous gene identification

Protein coding potential of each gene was estimated by Coding Potential Calculator (CPC) [36] and Coding Potential Assessment Tool (CPAT) [35] with default parameter. CPAT required human prebuilt hexamer frequency table and human prebuilt training model those CPAT utilized were both downloaded from https://sourceforge.net/projects/rna-cpat/files/v1.2.2/prebuilt_model/; and CPC’s reference protein dataset was downloaded from Uniref90 protein database. ORF regions were predicted by framefinder, which is implanted in CPC. Genes with positive coding potential defined by both softwares would be considered as protein coding genes. Then genes with coding potential went on pairwise orthologous gene detection. The best ORF regions were identified by Transdecoder (version 3.0.1) with homology to reviewed protein sequences via blast (version 2.4.0+) and pfam (release 31.0) searches.

Verified human peptide sequences were downloaded from Swiss-Prot database(http://www.uniprot.org/uniprot/?query=*&fil=reviewed%3Ayes+AND+organism%3A%22Homo+sapiens+%28Human%29+%5B9606%5D%22).

Pairwise orthologous genes between human and *M. fascicularis* were identified by InParanoid (version 4.1) [51] with default settings. Human protein ids were converted into ENSEMBL transcript and gene ids according to the Uniref database. Protein ids that related with more than one transcript ids would further selected by blastn (version 2.4.0+) on transcript level for best sequence match.

### Multi-tissues transcriptome analysis

RPKM (reads per kilobase of gene model per million mapped reads) of orthologous genes were calculated by Stringtie (with parameter: -e), limiting the processing of read alignments to only estimate the expression level of genes. The reference genome annotation of human was Gencode version 19, and for *M. fascicularis* RNA-seq datasets, new annotation was used as reference annotation. To render the data comparable across species and tissues, quantile normalization was used to scale data with preprocessCore package in R.

### Transcriptome complexity analysis

The average contribution of each orthologous gene to the total orthologous transcriptional output of each tissue was calculated following the process below:

1. He average expression of each orthologous gene was calculated across all samples of the same tissue
2. For each tissue, the average gene expression levels were sorted in decreasing order, and each value were divided by the sum of all orthologous genes’ average expression levels
3. The cumulative distribution of the contribution of each orthologous gene was plotted (Fig. 3a).

## Additional files

Additional file 1: **Figure S1.** a Summary of the number and source of *M. fascicularis*’ total RNA-seq samples. Orange: generated by our laboratory; Blue: generated by NHPRTR. b The length and count distribution of new *M. fascicularis* genome annotation, separately for each chromosome. (PDF 175 kb)

Additional file 2: **Table S1.** Information of all *M. fascicularis* RNA-seq data. (XLSX 13 kb)

Additional file 3: **Table S2.** Infromation of all protein data. (XLSX 10 kb)

Additional file 4: **Figure S2.** Examples of manual annotation. a Biological evidences supported an example of novel genes (CE_gene_1158). Due to the space limitation, part of transcripts and protein alignments had been showed. b Biological evidences supported an example of re-annotated genes (CE_gene_9026, Refseq gene name: rplp2). Due to the space limitation, protein alignments, part of transcripts and EST had not been showed. c Browser view of an example of novel genes (CE_gene_1158) by total RNA-seq of 10 samples. d Browser view of an example of re-annotated genes (CE_gene_9026, Refseq gene name: RPLP2) by total RNA-seq of 10 samples. (PDF 1636 kb)

Additional file 5: **Figure S3.** Quantitative measure of the comparison of different annotation versions. a Boxplot of the congruence of Ensemble *Macaca fascicularis* 5.0.91 annotation, NCBI *Macaca fascicularis* release 101 and the new annotation (manual) on gene level. b Density of the congruence of Ensemble *Macaca fascicularis* 5.0.91 annotation, NCBI *Macaca fascicularis* release 101 and the new annotation (manual) on exon level. (PDF 3225 kb)

Additional file 6: **Table S3.** Orthologous gene list. (XLSX 854 kb)

Additional file 7: **Table S4.** Information of Human RNA-seq data. (XLSX 13 kb)

Additional file 8: **Figure S4.** Comparability of tissue expression across human and *M. fascicularis*. a Pearson correlation coefficient matrix of different tissues, left panel shows human, right shows *M. fascicularis*. Blue means lowest correlation coefficient, orange means highest correlation coefficient. b Functional enrichment of cortex specifically high expressed genes. c Functional enrichment of pituitary and stomach specifically high expressed genes. (PDF 545 kb)

Additional file 9: **Table S5.** The details of each *M. fascicularis*. (XLSX 10 kb)

## Abbreviations

cDNA: Complementary DNA
CPAT: Coding Potential Assessment Tool
CPC: Coding Potential Calculator
EST: Expressed sequence tag
EVM: EVidenceModeler
PASA: Program to Assemble Spliced Alignments
PBMC: Peripheral Blood Mononuclear Cell
PCR: Polymerase chain reaction
RefSeq: The Reference Sequence
RNA-seq: RNA Sequencing
RPKM: Reads per kilobase of gene model per million mapped reads
