# Supplementary figures and images for: Improved Macaca fascicularis gene annotation reveals evolution of gene expression profiles in multiple tissues
Source: BMC Genomics. 2018 Nov 1;19:787. doi: 10.1186/s12864-018-5183-y (PMC6211470; doi:10.1186/s12864-018-5183-y)

**a**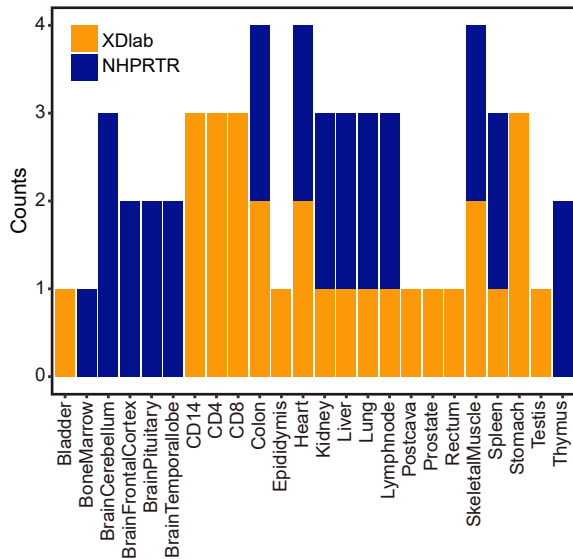**b**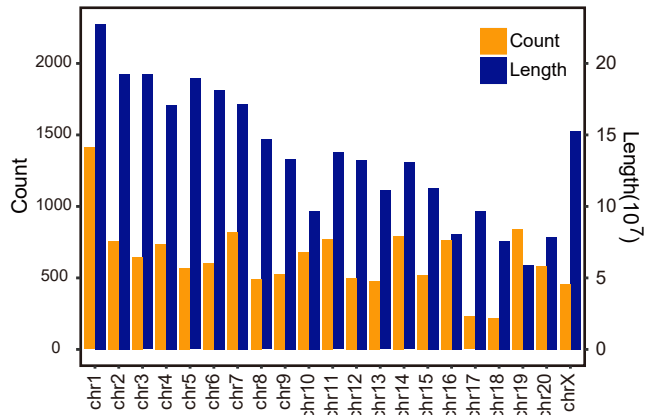

Supplement: Supplementary file 1 — Figure S1. a Summary of the number and source of M. fascicularis’ total RNA-seq samples. Orange: generated by our laboratory; Blue: generated by NHPRTR. b The length and count distribution of new M. fascicularis genome annotation, separately for each chromosome. (PDF 175 kb) [file 12864_2018_5183_MOESM1_ESM.pdf]

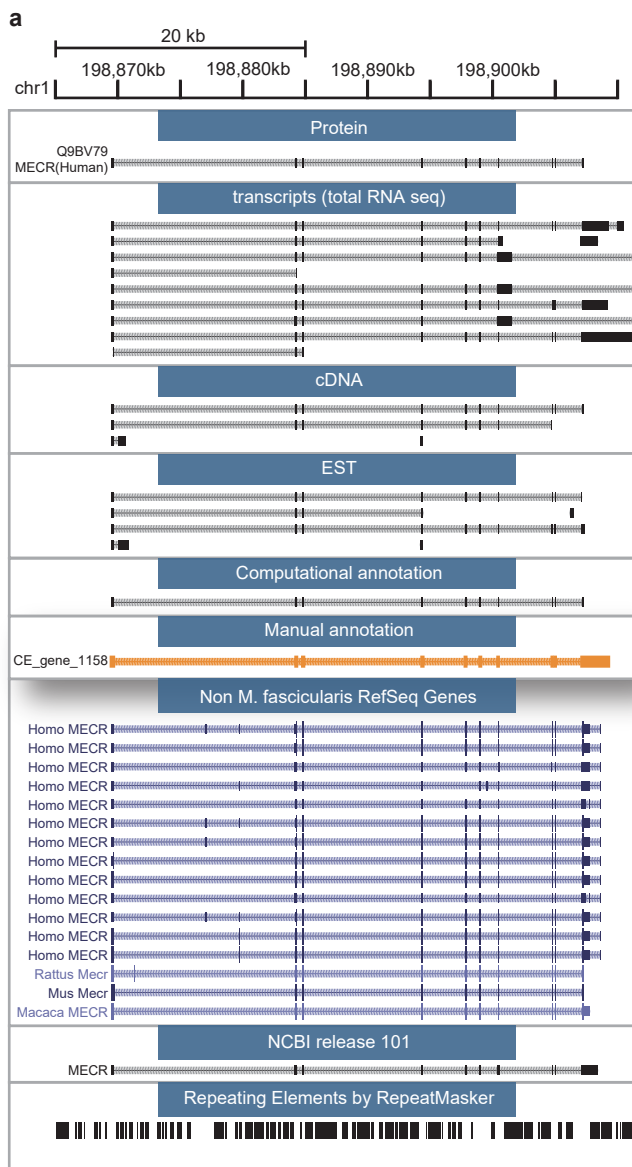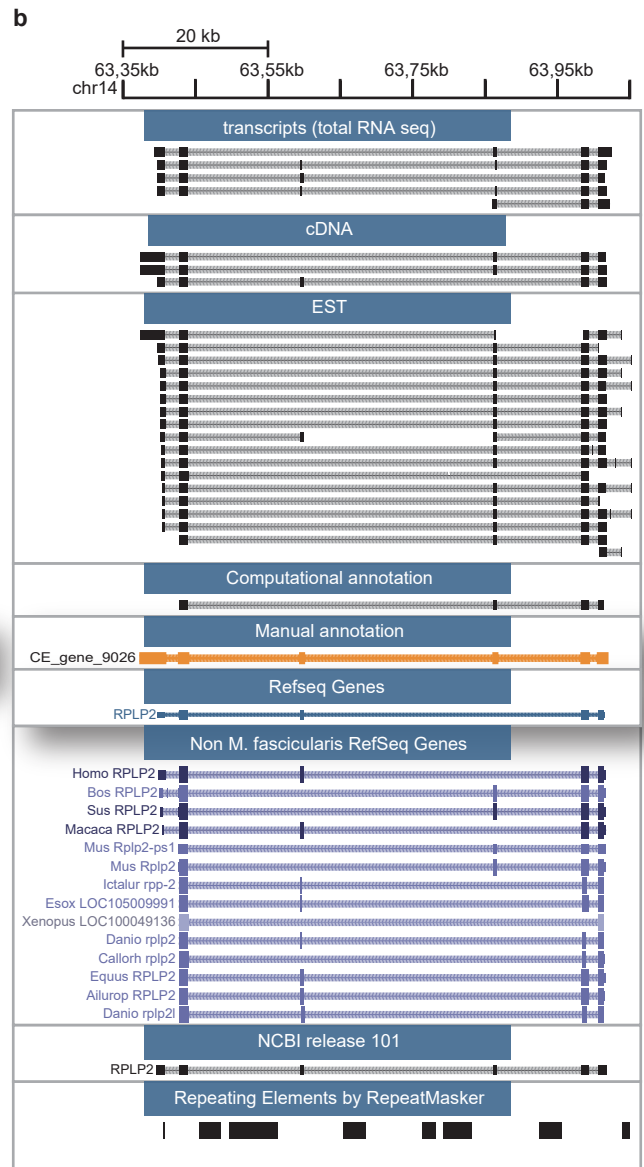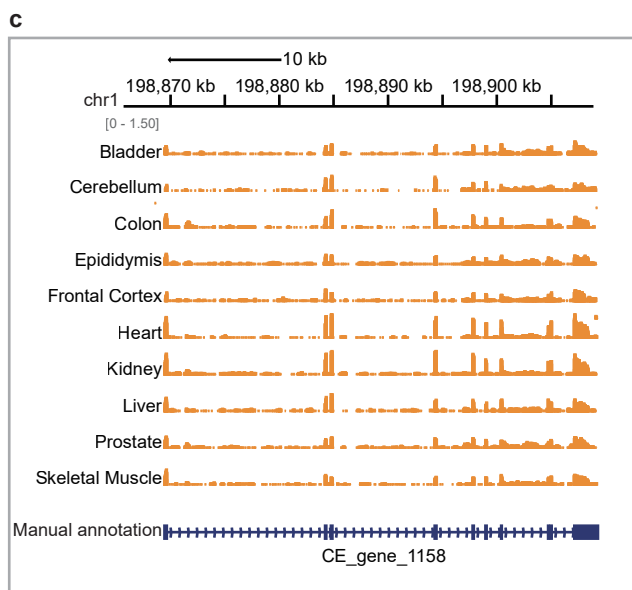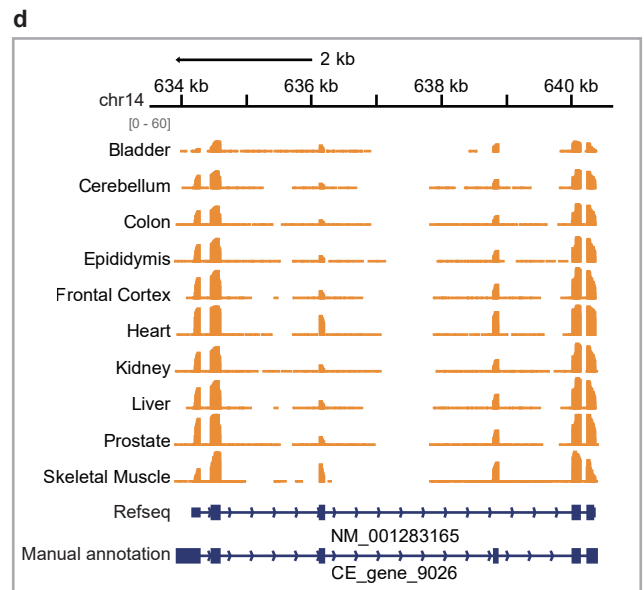

Supplement: Supplementary file 4 — Figure S2. Examples of manual annotation. a Biological evidences supported an example of novel genes (CE_gene_1158). Due to the space limitation, part of transcripts and protein alignments had been showed. b Biological evidences supported an example of re-annotated genes (CE_gene_9026, Refseq gene name: rplp2). Due to the space limitation, protein alignments, part of transcripts and EST had not been showed. c Browser view of an example of novel genes (CE_gene_1158) by total RNA-seq of 10 samples. d Browser view of an example of re-annotated genes (CE_gene_9026, Refseq gene name: RPLP2) by total RNA-seq of 10 samples. (PDF 1636 kb) [file 12864_2018_5183_MOESM4_ESM.pdf]

**a**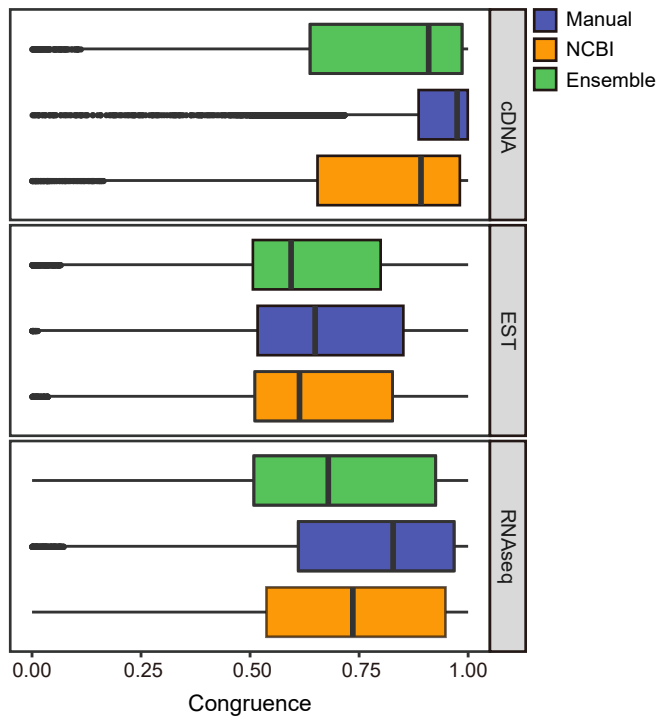**b**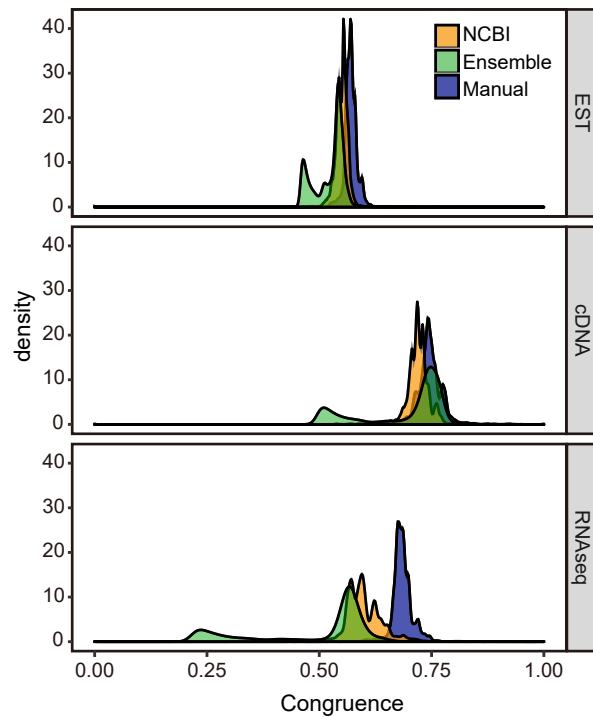

Supplement: Supplementary file 5 — Figure S3. Quantitative measure of the comparison of different annotation versions. a Boxplot of the congruence of Ensemble Macaca fascicularis 5.0.91 annotation, NCBI Macaca fascicularis release 101 and the new annotation (manual) on gene level. b Density of the congruence of Ensemble Macaca fascicularis 5.0.91 annotation, NCBI Macaca fascicularis release 101 and the new annotation (manual) on exon level. (PDF 3225 kb) [file 12864_2018_5183_MOESM5_ESM.pdf]

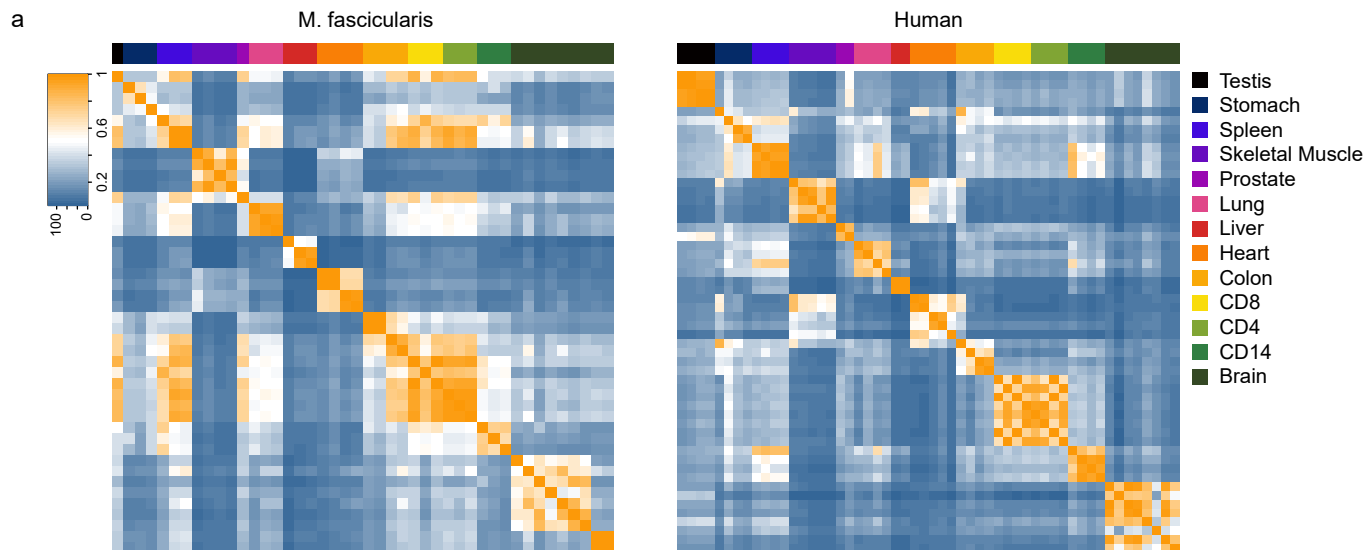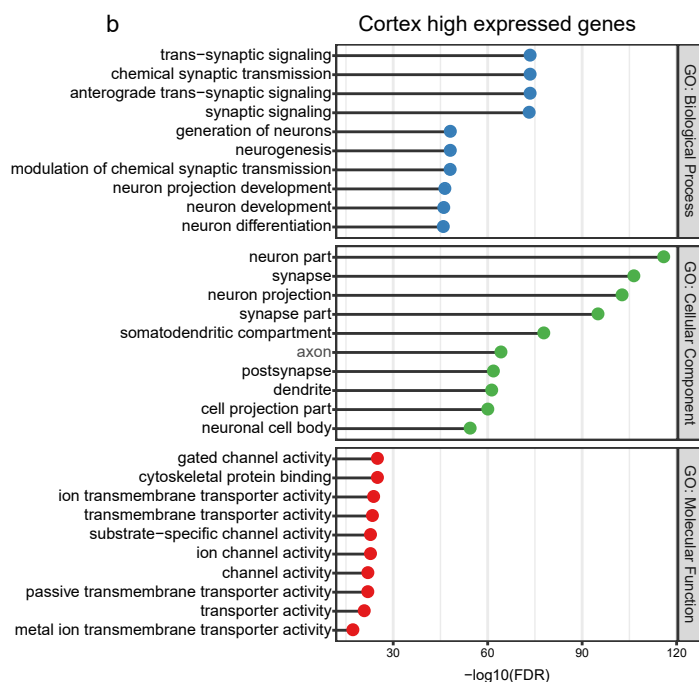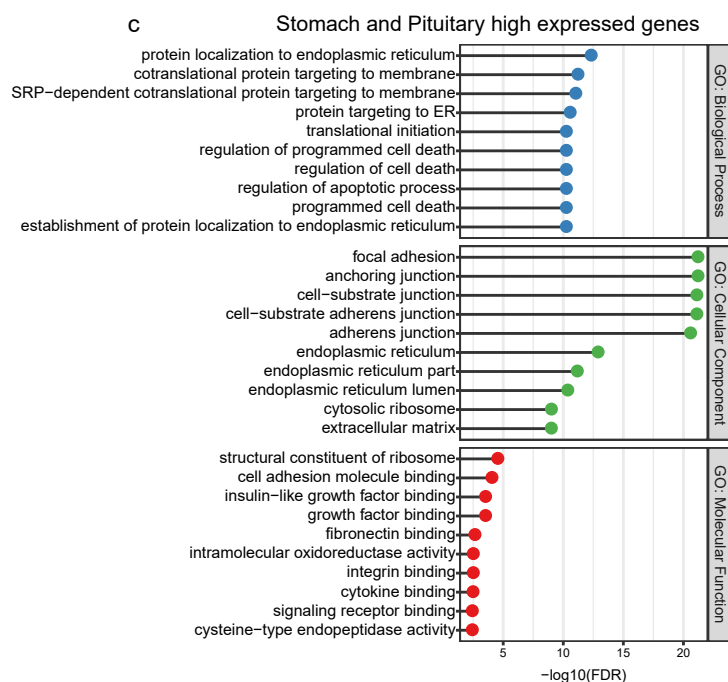

Supplement: Supplementary file 8 — Figure S4. Comparability of tissue expression across human and M. fascicularis. a Pearson correlation coefficient matrix of different tissues, left panel shows human, right shows M. fascicularis. Blue means lowest correlation coefficient, orange means highest correlation coefficient. b Functional enrichment of cortex specifically high expressed genes. c Functional enrichment of pituitary and stomach specifically high expressed genes. (PDF 545 kb) [file 12864_2018_5183_MOESM8_ESM.pdf]
